# Phylogenetic analysis of xylan-degrading hemicellulases from *Talaromyces* and *Penicillium*: taxonomic and ecophysiological implications

**DOI:** 10.3389/fmicb.2026.1818172

**Published:** 2026-07-15

**Authors:** Ana Pozo-Rodríguez, Juan A. Méndez-Líter, Alicia Prieto, María Jesús Martínez, Jorge Barriuso

**Affiliations:** Microbial Systems and Protein Engineering Group, Department of Biotechnology, Center for Biological Research Margarita Salas, Spanish National Research Council (CIB-CSIC), Spanish National Research Council (CIB-CSIC), Madrid, Spain

**Keywords:** biomass valorization, fungal hemicellulases, *Penicillium*, phylogenetic analysis, *Talaromyces*

## Abstract

Xylan degradation in filamentous fungi relies on a diverse repertoire of hemicellulases whose evolutionary history and taxonomic distribution remain incompletely resolved. Here, we performed a comparative phylogenomic survey of 585 putative xylan-active CAZymes belonging to 10 CAZy families (GH3, GH10, GH11, GH43, GH51, GH54, GH62, GH67, CE1 and CE5) from 37 genomes of *Talaromyces* and *Penicillium* species. Sequences were classified and phylogenetically compared to evaluate their distribution across taxa and reported ecological groups. The phylogenetic reconstructions revealed lineage-associated expansions, conserved evolutionary clusters containing experimentally characterized enzymes, and family-dependent diversification patterns. *Penicillium* species displayed heterogeneous enzyme repertoires consistent with their broad ecological occurrence, with specific clades co-occurring with saprophytic or pathogenic lifestyles and with plant biomass-rich environments. On the other hand, *Talaromyces* exhibited comparatively homogeneous profiles congruent with its narrower lifestyle range. Multivariate clustering of CAZyme repertoires recapitulated these taxonomic and lifestyle-associated distribution patterns. These results provide a curated evolutionary framework for interpreting xylan-active CAZyme diversity in these genera and identify candidate enzymes for future functional characterization and biotechnological exploration.

## 1 Introduction

Hemicellulose is the second most abundant component of lignocellulosic biomass, consisting of various polysaccharides with diverse structures (Silva et al., 2024). These heteropolysaccharides differ in the types and the proportions of their constituent monosaccharides, backbone composition, branching patterns, and types of glycosidic linkages. Hemicelluloses are categorized into four types based on the predominant monosaccharide in their polymeric chain: xylans, mannans, xyloglucans, and mixed-linkage β-glucans. Xylan, the most prevalent hemicellulose in dicotyledons and cereal grains, has a β-(1,4)-D-xylopyranose backbone with various side chains containing arabinose (arabinoxylans), glucuronic acid (glucuronoxylan) or a mixture of both residues (arabinoglucuronoxylans or glucuronoarabinoxylans) (Smith et al., 2017). Mannans or galactomannans are composed of a β-(1,4)-D-mannopyranose main chain partially branched. Xyloglucans are characterized by a cellulose-like β-(1,4)-D-glucopyranose backbone with α-xylopyranose side chains. Finally, mixed-linkage β-glucans feature a linear backbone of β-(1,3) and β-(1,4)-D-glucopyranose units.

The complex structure and chemical diversity of hemicelluloses require the coordinated action of multiple types of hemicellulases. Most of them are glycosyl hydrolases (GH) with different specificities, but some carbohydrate esterases (CE) are also relevant accessory enzymes for effective degradation (Álvarez et al., 2016). In particular, the hemicellulases involved in xylan breakdown are: (i) endoxylanases, that cleave the internal linkages of xylan backbone mainly classified in families GH10 and GH11 in the CAZy database (Drula et al., 2022); (ii) β-xylosidases, that hydrolyze xylo-oligosaccharides into xylose (mostly from GH3 and GH43 families); (iii) arabinofuranosidases that remove arabinose side chains (predominantly GH51, GH54, GH62); (iv) *α*-glucuronidases that eliminate glucuronic acid ramifications (mainly GH67 and GH115); and (v) acetyl-xylan esterases (primarily grouped in CE1 and CE5) and feruloyl esterases (CE1), accessory enzymes that remove acetyl and feruloyl groups from xylan, making polysaccharides easier to degrade. CAZy family classification is based on sequence similarity and structural features and does not necessarily imply experimentally validated enzymatic activity. These and other hemicellulases (i.e., mannanases, galactosidases, fucosidases) can be obtained from a broad variety of microorganisms, but Ascomycetes from the genera *Trichoderma*, *Aspergillus*, *Penicillium* and *Talaromyces* are widely regarded as prominent producers of high yields of a broad spectrum of hemicellulases (Polizeli et al., 2005; Srivastava et al., 2018). Additionally, fungal hemicellulases are typically secreted, and tend to be more stable, more active at acidic pH, and more thermostable than their bacterial counterparts. Secretion is nonetheless not universal, and some activities may remain cell-wall-associated or be coupled to oligosaccharide-uptake systems, analogous to the ‘selfish’ xylo-oligosaccharide assimilation strategies described in bacteria (Zhang et al., 2014; Hao et al., 2021). Thus, the advantages of fungal hemicellulases make them attractive biocatalysts for multiple industrial sectors (Kuhad et al., 2011). For example, they improve the production of bioethanol and other bioproducts, contribute to more efficient processes and better product quality in the pulp and paper industry, and are used to enhance the nutritional value and functionality of plant-based ingredients in the food sector (Shrivastava, 2020). Given their industrial relevance, a comparative overview of their sequence diversity and taxonomic distribution can facilitate the identification of candidates for future biochemical characterization and biotechnological exploration. Due to their remarkable enzymatic capabilities, *Penicillium* and *Talaromyces* species play a key role in decomposition of complex organic materials in nature (Méndez-Líter et al., 2021). These genera are closely related phylogenetically and, before the era of molecular phylogeny, *Talaromyces* was a subgenus of *Penicillium* that later split off as an independent genus (Samson et al., 2011). Both include cosmopolite and ecologically versatile species, most of them saprophytic, thriving in soil, decaying plant material, and indoor settings, although some species have been reported as opportunistic pathogens under specific host conditions (Houbraken et al., 2010; Visagie et al., 2014). *Penicillium* encompasses over 500 species, many of them industrially renowned in the food sector and as producers of secondary metabolites (Yilmaz et al., 2014; Houbraken et al., 2020) while *Talaromyces* contains more than 170 species and is mainly known for its arsenal of cellulolytic and hemicellulolytic enzymes (Prieto et al., 2021; Pozo-Rodríguez et al., 2025). Taxonomically, *Penicillium* and *Talaromyces* are divided into different infrageneric groups (subgenera and sections), some of which can be associated with specific ecological lifestyles (Houbraken et al., 2016). According to the last classifications, *Penicillium* species are distributed into two subgenera and 32 sections (e.g., *Fasciculata*, *Penicillium, Robsamsonia*, *Roquefortorum*), while *Talaromyces* species are grouped into 8 sections (e.g., *Helici*, *Islandici*, *Talaromyces*, *Trachyspermi*) (Da Sun et al., 2020). However, as the number of newly discovered species continues to increase, additional sections are likely to be established in the future. In addition, fungal taxonomy continues to evolve as new molecular data emerge, often leading to the reclassification of species. This is the case of *Penicillium occitanis,* one of the species studied in the current work. Despite it is not presently ascribed to any of the sections defined within *Penicillium,* phylogenetic data suggest its relatedness with *Talaromyces* and recommend reclassification within the *Talaromyces* clade (Bravo-Ruiz et al., 2017).

Considering their widespread distribution, the phylogenetic analysis of their xylan-active repertoires may reveal patterns of diversification associated with taxonomic history and reported ecological occurrence (de Vries and Visser, 2001; Li et al., 2022). In this context, it is known that plant-pathogenic species often exhibit high levels of hemicellulases to break down the host’s cell wall and enable colonization (Lorrai and Ferrari, 2021) while saprophytic fungi produce a broader spectrum of very specific hydrolases for efficient degradation of complex polysaccharides (Shah et al., 2016). In addition, some studies related the genetic diversity of *Penicillium* and *Talaromyces* species in environments like soil, freshwater, and clinical samples, with their ecological roles and potential applications (Tsang et al., 2018). Similarly, the relationship between the phylogeny/taxonomy of *Penicillium* species and their ecological role, lifestyle, and carbohydrate-active enzyme (CAZymes) profile was also analyzed (Barrett et al., 2020).

Currently, massive sequencing of fungal genomes provides an increasing number of gene sequences potentially encoding CAZymes (Lange et al., 2021), allowing their comparative study. Thus, taxonomic and phylogenetic analyses of the vast amount of new data can provide a framework to examine how hemicellulase diversity is distributed across fungal taxa and ecological descriptions (de Vries and Visser, 2001; Barrett et al., 2020; Li et al., 2022; de la Fuente et al., 2024).

In this work, we present a comprehensive list of genes from *Penicillium* and *Talaromyces* species, encoding putative hemicellulose-active enzymes inferred from CAZy family assignment, retrieved from predicted proteomes derived from publicly available genome assemblies. The enzymes were classified into their corresponding families, and their phylogenetic relationships were analyzed, comparing their presence and distribution across taxonomic groups and reported ecological categories. The main aim of this study is therefore to provide a detailed phylogenomic analysis of xylan-degrading hemicellulases from *Talaromyces* and *Penicillium*, characterizing the distribution, domain features and evolutionary patterns of CAZy families central to fungal xylanolysis. By integrating phylogenies with lifestyle metadata and comparative analyses of enzyme repertoires, we seek to explore whether diversification patterns are consistent with taxonomic relationships and reported ecological occurrence. Moreover, unlike previous secretome and family-level comparisons of fungal CAZymes (Barrett et al., 2020; Li et al., 2022), which assess overall enzyme categories, here we resolve the intra-family diversification and module architecture (CBM content and subfamily assignment) of the complete xylanolytic system across two sister genera, identifying specific candidate clades and lineage-associated expansions that constitute concrete leads for enzyme prospecting. To our knowledge, no comparative evolutionary framework has so far focused specifically on the complete xylanolytic system across these two sister genera. As Talaromyces was historically classified within Penicillium, their joint analysis offers an informative setting in which to examine how xylanolytic repertoires diverge between closely related lineages following taxonomic separation. While the identification of uncharacterized hemicellulases has clear potential for future biotechnological exploration, the core contribution of this work is to establish a curated comparative evolutionary framework that can guide future functional and applied studies.

## 2 Methods

### 2.1 Sequence retrieval and dataset curation

Protein sequences from the genomes of *Penicillium* and *Talaromyces* species were retrieved from the MycoCosm Fungal Portal^1^ and Uniprot^2^ databases. Public theoretical proteomes were mainly selected for MycoCosm, taking from Uniprot only those entries that were not present in the other database. Proteomes with unknown species or unrealistic protein counts were discarded. In total, 37 proteomes were used in this study (Supplementary Table SI).

BLASTp was used to search the genomes for GH (glycosyl hydrolases) and CE (carbohydrate esterases) active on xylan, using different model sequences (Supplementary Table SII) for the queries: GH10 and GH11 endo-β-1,4-xylanases (EC 3.2.1.8), GH3 and GH43 β-xylosidases (EC 3.2.1.37), GH51, GH54 and GH62 *α*-L-arabinofuranosidases (EC 3.2.1.55), GH67 α-glucuronidases (EC 3.2.1.139), CE1 and CE5 acetylxylan esterases (EC 3.1.1.72), and CE1 feruloyl esterases (EC 3.1.1.73). GH30 enzymes, in particular the fungal glucuronoxylan-specific xylanases of subfamily 7, also participate in xylan (specifically glucuronoxylan) degradation but were not included in the present survey, which focused on the canonical xylanolytic families for which well-supported model query sequences were available for *Penicillium* and *Talaromyces*. Given their role in glucuronoxylan hydrolysis, GH30 enzymes represent a natural extension of the framework presented here for future analyses (Puchart et al., 2021). When possible, sequences were chosen from *Penicillium* and *Talaromyces* species or a closely related fungus (Supplementary Table SII). For the BLASTp searches, an e*-*value cutoff of 1e*-*5 was applied and only the catalytic domains predicted by dbCAN (Zhang et al., 2018) of the query sequences were considered.

The resulting datasets obtained with the BLASTp searches were analyzed with AliView v.1.28 (Larsson, 2014) to remove low quality sequences due to inaccurate annotation. This is the case of very short or very long sequences and sequences with big gaps. Only publicly available assembled and annotated genomes were considered. Sequences removed during curation corresponded to incomplete gene models or truncated predictions, and their exclusion was applied uniformly across datasets to minimize annotation artifacts rather than to bias gene counts. Finally, putative signal peptides (SP) and carbohydrate binding modules (CBM) of the sequences were predicted using SignalP v. 5.0 (Almagro Armenteros et al., 2019) and dbCAN servers (Zhang et al., 2018), respectively. The CAZy family to which the sequences belonged was determined using InterProScan (Blum et al., 2021). Sequences were renamed with their CAZy family followed by their JGI or Uniprot accession number, the abbreviation of their fungal genus and species, and the strain number if there was more than one representative of the same species. The resulting dataset consisted of 585 sequences from *Penicillium* and *Talaromyces* proteomes, categorized into 10 hemicellulase families (Supplementary Table SIII). Functional annotation was inferred from CAZy family assignment and conserved catalytic domains identified by InterProScan. No biochemical validation was performed; therefore, all activities mentioned throughout the manuscript should be considered predicted based on sequence homology.

### 2.2 Phylogenetic analyses of hemicellulases sequences

Phylogenetic analyses were carried out for each family of enzymes active on xylan. First, signal peptides (SPs) and carbohydrate-binding modules (CBMs) were removed from the sequences, as they are known to align poorly due to their heterogeneity and low conservation (Barruetabeña et al., 2019). Multiple sequence alignments were then performed using the MAFFT online server^3^ with default settings (Katoh et al., 2019). The alignments generated were subsequently trimmed with trimAl v. 1.3 (Capella-Gutiérrez et al., 2009) selecting the gappyout method to delete poorly aligned regions caused by, for instance, incorrectly annotated introns. The most suitable evolution models for the sets of sequences were checked using ProtTest 2.4 v. (Abascal et al., 2005) among 56 empirical models of evolution. Maximum-Likelihood trees were then built with RAxML v. 8 (Stamatakis, 2014) using 100 bootstrap replicates and the following models of evolution suggested by ProtTest: PROTGAMMAWAG for GH10 and GH11 endoxylanases, GH43 β-xylosidases, GH54 and GH62 arabinofuranosidases and CE1 acetylxylan esterases and feruloyl esterases, PROTGAMMALG for GH3 β-xylosidases, GH51 arabinofuranosidases and GH67 *α*-glucuronidases, and PROTGAMMAJTT for CE5 acetylxlan esterases. Finally, the trees were visualized and rooted with FigTree v. 1.4.2.^4^ For tree rooting we included in the datasets of each hemicellulase family one characterized sequence from CAZy belonging to far-related fungi or bacteria. Phylogenetic trees were then studied to examine diversification patterns within each CAZy family and to compare clade distribution across reported ecological categories. Lifestyle information for each species was compiled from taxonomic monographs and primary literature sources cited in Table I. Species were assigned to descriptive ecological categories based on their most frequently reported habitat or interaction. These categories were used exclusively as comparative metadata to contextualize phylogenetic and repertoire patterns.

**Table I:** Taxonomy and habitat of the fungi studied in this work.

Fungus (and its abbreviation)	Taxonomy section	Lifestyle
*Penicillium*		
*Penicillium antarcticum* IBT 31811 (Pant)	Sect. *Canescentia*	Fallen leaves, plant soil
*Penicillium arizonense* CBS 141311 (Pari)		
*Penicillium chrysogenum* Wisconsin 54-1255 (Pchry_Wisconsin54-1255)	Sect. *Chrysogena*	Dry habitats, indoor environments
*Penicillium chrysogenum* P2niaD18 (Pchry_P2niaD18)		
*Penicillium flavigenum* IBT 14082 (Pfla)		
*Penicillium nalgiovense* FM193 (Pnal)		
*Penicillium steckii* IBT 24891 (Pste)	Sect. *Citrina*	Grass soil, agricultural soil
*Penicillium decumbens* IBT 11843 (Pde)	Sect. *Exilicoulis*	Plant soil, fallen leaves
*Penicillium camemberti* FM 013 (Pcam)	Sect. *Fasciculata*	Stored or manufactured food, fruits, cereals, vegetables
*Penicillium freii* DAOM 242723 (Pfr)		
*Penicillium nordicum* DAOMC 185683 (Pnord)		
*Penicillium polonicum* IBT 4502 (Ppol)		
*Penicillium solitum* IBT 29525 (Psol)		
*Penicillium brasilianum* MG11 (Pbra_MG11)	Sect. *Lanata-divaricata*	Grass soil, agricultural soil
*Penicillium brasilianum* LaBioMMi 136 (Pbra_LaBioMMi 136)		
*Penicillium oxalicum* 114-2 (Pox)		
*Penicillium subrubescens* CBS 132785 (Psub)		
*Penicillium digitatum* PHI26 (Pdi_PHI26)	Sect. *Penicillium*	Plant pathogens (apples, citrus)
*Penicillium digitatum* Pd1 (Pdi_Pd1)		
*Penicillium italicum* PHI-1 (Pita)		
*Penicillium expansum* CMP-1 (Pex_CMP1)		
*Penicillium expansum* MD-8 (Pex_MD8)		
*Penicillium coprophilum* IBT 31321 (Pcop)	Sect. *Robsamsonia*	Dung, dry cereals and seeds
*Penicillium griseofulvum* PG3 (Pgri)		
*Penicillium vulpinum* IBT 29486 (Pvul)		
*Penicillium roqueforti* FM164 (Pro)	Sect. *Roquefortorum*	Soil, plants, fruits, food (i.e., cheese)
*Talaromyces*		
*Talaromyces islandicus* WF3812 (Tis)	Sect. *Islandici*	Plant soil, biomass waste
*Talaromyces rugulosus* W13939 (Tru)		
*Talaromyces stipitatus* ATCC 10500 (Tst)	Sect. *Talaromyces*	
*Talaromyces amestolkiae* CIB (Tam)		
*Talaromyces cellulolyticus* Y-94 (Tce)		
*Talaromyces marneffei* ATCC 18224 (Tma_ATCC18224)		
*Talaromyces marneffei* PM1 (Tma_PM1)		
*Talaromyces marneffei* 11CN-03-130 (Tma_11CN-03-130)		
*Talaromyces atroroseus* IBT 11181 (Tat)	Sect. *Trachyspermi*	
*Penicillium occitanis* CL100 (Poc_CL100)^1^	-	
*Penicillium occitanis* CT1 (Poc_CT1)^1^		

1 This species is currently considered as incertae sedis, but several molecular studies provide overwhelming data that suggest that it should be renamed and transferred to Talaromyces.

### 2.3 Multivariate analysis of the hemicellulases battery and fungal species

A matrix with the number of enzymes grouped in each hemicellulases family and each *Penicillium* or *Talaromyces* proteome was created from Table SIII. This matrix was used for multivariate analysis using the heatmap() function of R v. 4.1.3^5^ using as default parameters the Euclidean distance method and the Complete linkage method. The results were represented in a heatmap, in which the resulting clustering was inspected to qualitatively compare enzyme repertoire patterns across taxonomic and ecological categories.

## 3 Results and discussion

This study provides an integrated analysis of the xylanolytic enzymes identified in the genomes of 37 species from *Penicillium* and *Talaromyces*. Each sequence was assigned to its corresponding CAZy family and compared phylogenetically to examine diversification patterns across fungal taxa and reported ecological categories. Before deepening into the phylogenomic analyses, we reviewed the available literature on the habitat and lifestyle of the species analyzed, to contextualize their enzymatic profiles. Observed distribution patterns are discussed in light of available ecological descriptions and previously reported functional data.

### 3.1 Analysis of the lifestyle of the selected fungi

Table I offers detailed information on the *Penicillium* (Visagie et al., 2014; Houbraken et al., 2016) and *Talaromyces* species (Yilmaz et al., 2014) studied and their ecological niches. In-depth analysis of these data provided multiple insights into the habitats and enzymatic capabilities of the species included in this study, which were organized into nine descriptive ecological categories for comparative purposes, represented with nine different colours in Table I. These categories reflect recurrent habitat types (e.g., soil, plant material, fruit-derived substrates, food environments, indoor niches or pathogenic associations) and provide a framework for interpreting variation in their xylan-degrading repertoires.

Overall, *Penicillium* species display marked ecological heterogeneity, occupying niches that range from agricultural soils and decaying vegetation to fruits, stored food products, indoor environments and plant pathogenic interactions. This ecological breadth may be reflected in the heterogeneity of hemicellulase repertoires observed across the genus, as species adapted to distinct substrates often show expansions in different CAZy families involved in plant cell wall degradation.

In contrast, *Talaromyces* species exhibit a more restricted ecological range, with most representatives associated with soil, biomass waste and decomposing plant material. This more consistent ecological profile is accompanied by comparatively homogeneous CAZyme repertoires in the sampled genomes, which is in agreement with the high xylanolytic and cellulolytic capacities previously reported for several members of this genus (Da Sun et al., 2020).

This ecological classification serves as a baseline for interpreting the phylogenetic patterns observed in subsequent sections. Differences in lifestyle and substrate specialisation between *Penicillium* and *Talaromyces* are reflected in the contrasting heterogeneity of their enzyme repertoires, as developed in the following sections. It should be noted that these ecological categories are based on literature descriptions and do not represent quantitatively tested lifestyle classifications. Therefore, associations discussed below should be interpreted as qualitative observations. This study does not aim to statistically test ecological associations but to provide a comparative phylogenomic overview of CAZyme diversification patterns across taxa.

### 3.2 Phylogenetic analysis of xylan-active enzymes from *penicillium* and *Talaromyces*

**Figure 1:**
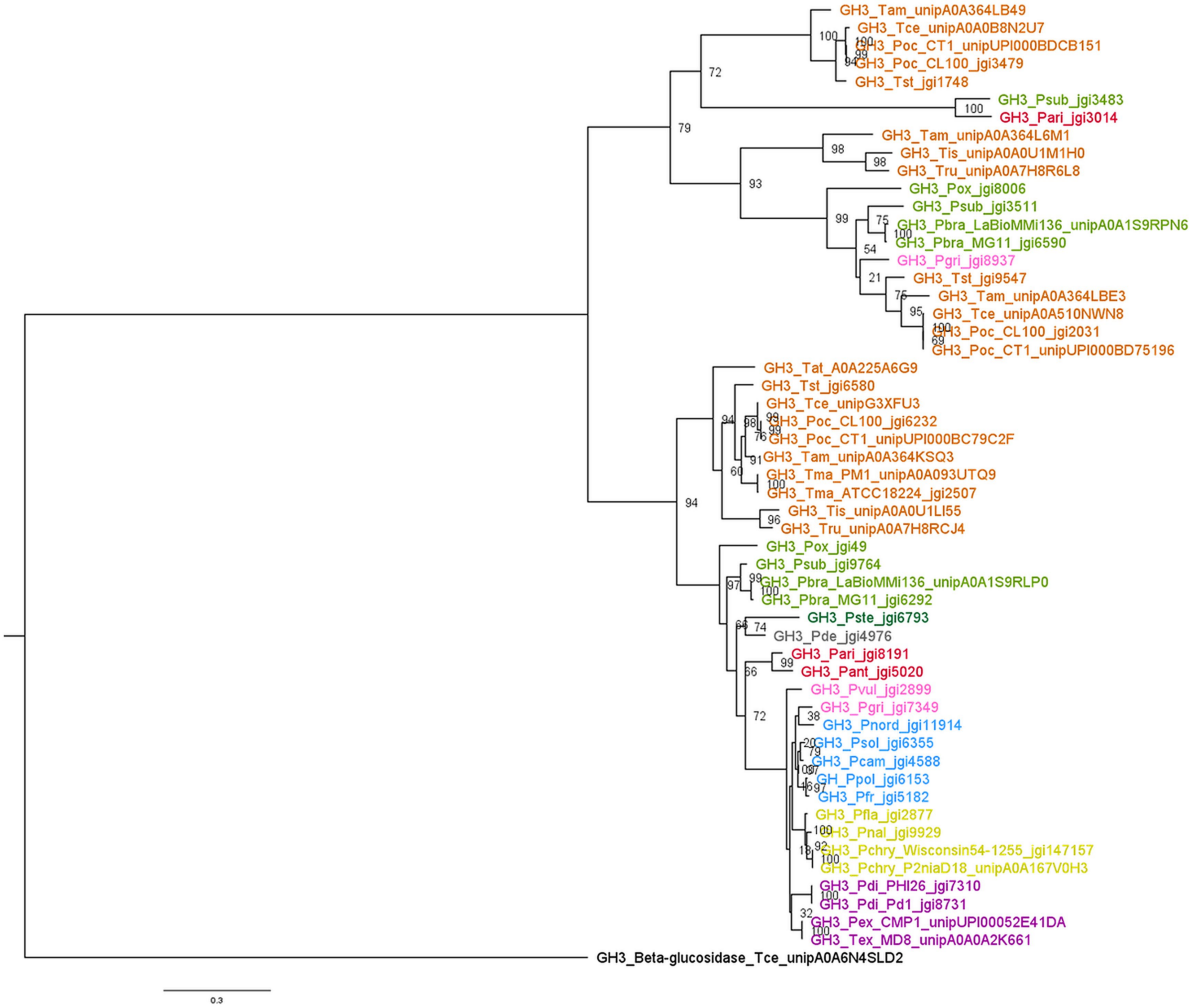
Phylogenetic tree of GH3 β-xylosidases from *Penicillium* and *Talaromyces*. The root sequence is a GH3 β-glucosidase from *Talaromyces cellulolyticus* Y-94.

**Figure 2:**
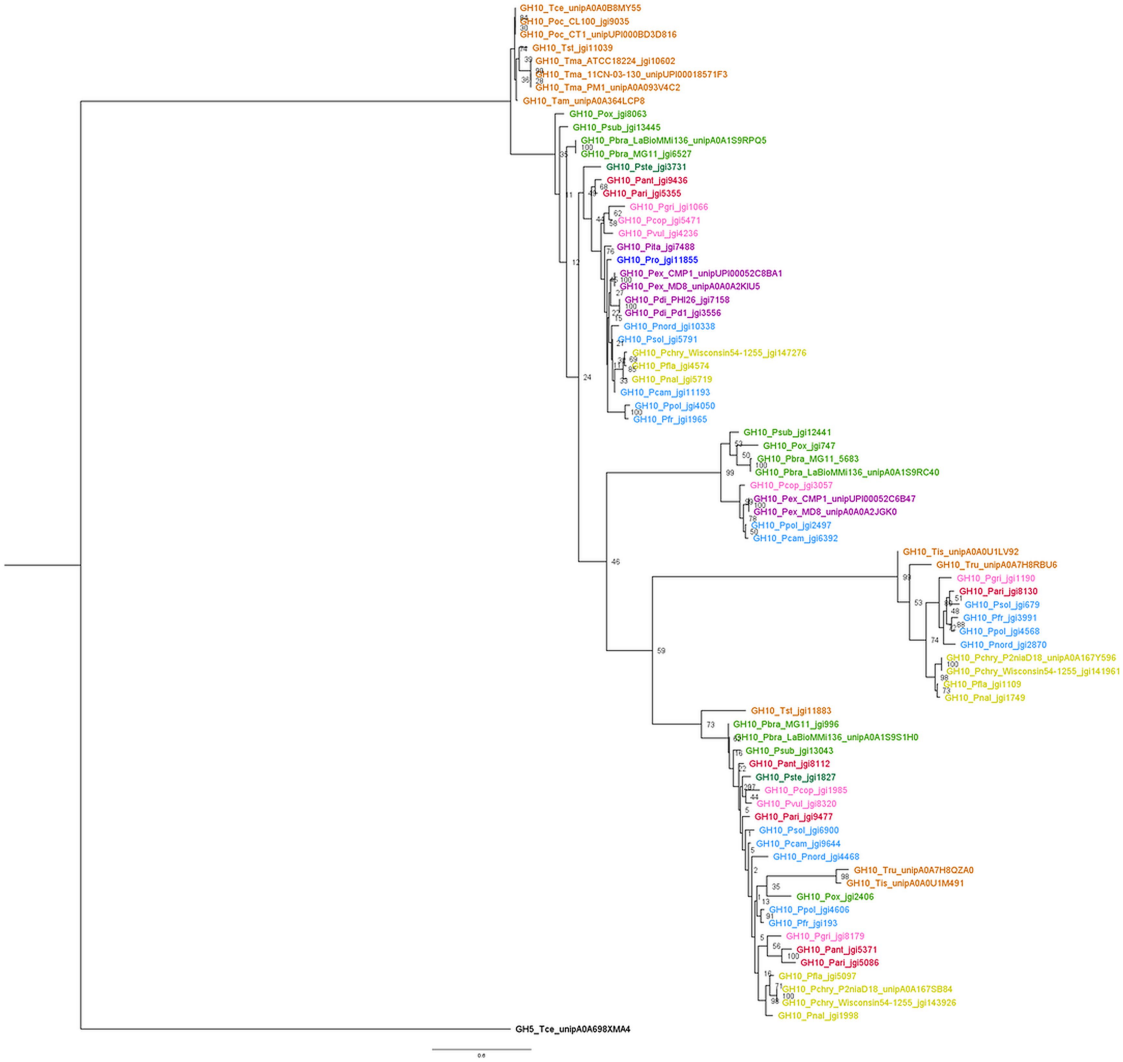
Phylogenetic tree of GH10 endoxylanases from *Penicillium* and *Talaromyces*. The root sequence is a GH5 endoglucanase from *Talaromyces cellulolyticus* Y-94.

**Figure 3:**
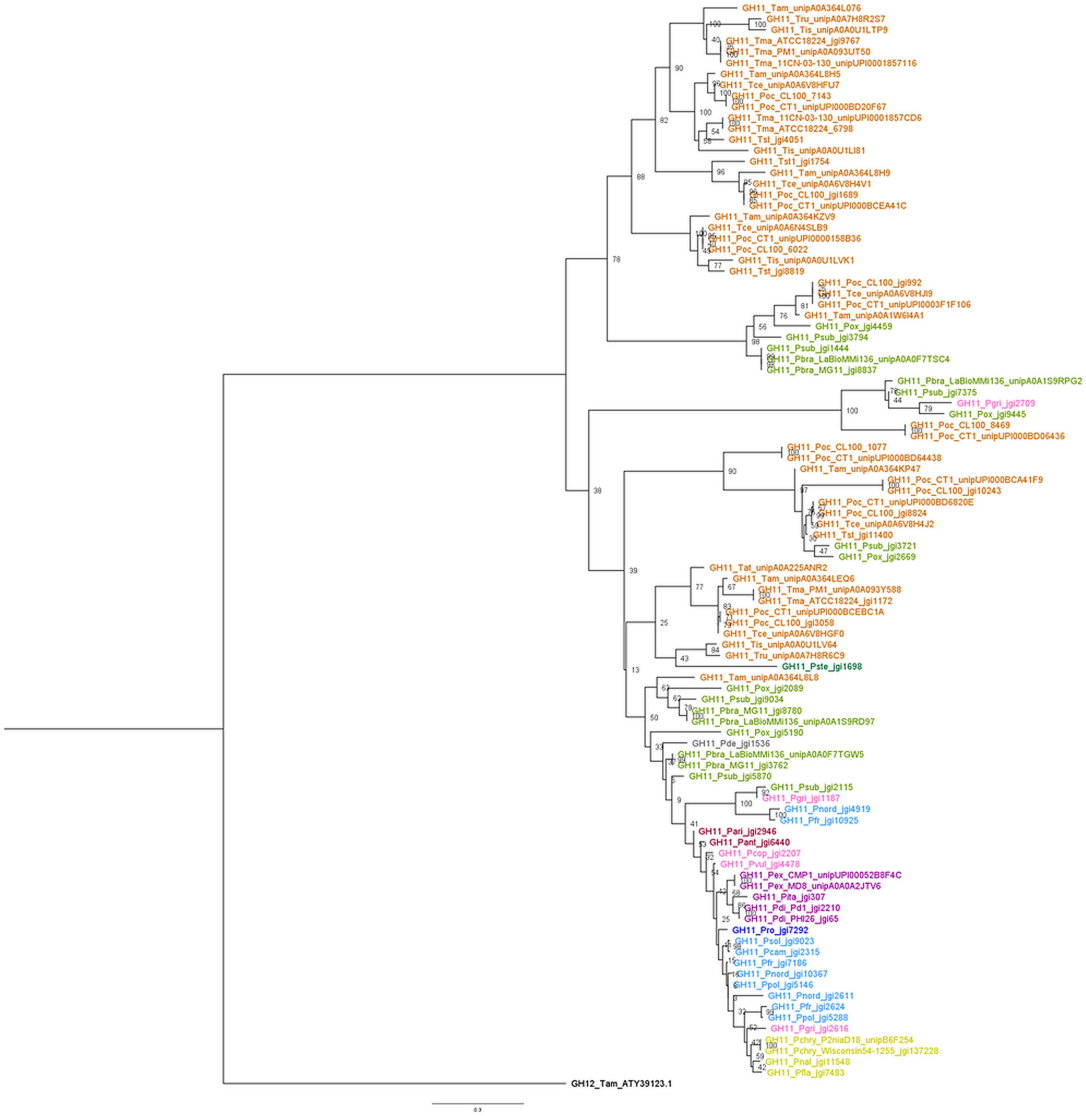
Phylogenetic tree of GH11 enzymes from *Penicillium* and *Talaromyces*. The root sequence is a GH12 endoglucanase from *Talaromyces amestolkiae* CIB.

To provide a clear overview of the xylan-degrading machinery encoded in the analyzed genomes, the following sections outline the main features of each identified CAZy family, as well as its phylogenetic analysis. The phylogenetic trees of xylan-active enzymes (Figures 1-3; Supplementary Figures S1–S7) were built based only on the catalytic domain. Some sequences also contained additional accessory modules such as predicted CBMs, which were noted when present but not used as classification criteria. The results reveal diversification patterns that are consistent with differences in taxonomic distribution and reported ecological occurrence, according to their roles in degrading xylan or other hemicelluloses. The appearance of enzymes in multiple clusters throughout the phylogenetic trees revealed significant evolutionary diversification within these genera. The divergence observed within clades may reflect adaptive evolution to specific environmental conditions, potentially giving rise to subfamilies within a GH group. Moreover, enzymes that do not cluster with others could represent unique evolutionary trajectories or distinct sequence features. These outlier sequences represent candidates for future biochemical characterization. The number of sequences of hemicellulases active on xylan in each family is presented in Supplementary Table SIII. Although some gene models had not been annotated as hemicellulases in the respective species, all retrieved sequences fall within well-established CAZy families. Their phylogenetic placement therefore provides family-level context rather than evidence of functional or discovery novelty.

### 3.3 Phylogenetic analysis of β-xylosidase families GH3 and GH43

β-Xylosidases catalyze the hydrolysis of short xylooligosaccharides into monomeric xylose units (Knob et al., 2010) Among filamentous fungi, they are mostly classified within the GH3 family, which is one of the most widely represented in fungal genomes. However, despite efforts to subdivide the GH3 family into subfamilies, a robust subfamily classification is currently not available. The structure of GH3 enzymes generally consists of a catalytic domain and a C-terminal fibronectin-like domain (FNIII; Sidar et al., 2020). They lack CBMs and are extracellular, having a signal peptide to direct the protein to the secretory pathway.

According to the phylogenetic tree presented in Figure 1, these enzymes are distributed in two major clades. The first one (upper) primarily comprises species from *Talaromyces* sect. *Talaromyces* and *Penicillium* sect. *Lanata-di*var*icata*, both mainly found in vegetation-rich soils (Table I). The second cluster is much more heterogeneous, including species from nine different sections related to a wider variety of lifestyles. Plant pathogenic species cluster in the lowest clade, apart from the saprophytes.

It can be noticed that fungi from *Talaromyces* sect. *Talaromyces* and *Penicillium* sect. *Lanata-divaricata* presented a higher diversity of β-xylosidases than the other sections (Supplementary Table SIII), which may represent lineage-specific diversification within this family. Notably, GH3 β-xylosidases are not functionally homogeneous: characterized members differ markedly in substrate affinity, tolerance to xylose inhibition, transglycosylation capacity, thermostability and pH optima (Knob et al., 2010). Consequently, the lineage-specific variants identified here represent candidates whose distinct biochemical properties could be exploited to optimize targeted processes; resolving their specificity could therefore play a decisive role in tailoring biocatalysts to particular industrial conditions, such as the efficient saccharification of xylo-oligosaccharides into xylose.

On the other hand, GH43 enzymes usually have modular structures, can have CBMs, and many of them exhibit both β-xylosidase and α-L-arabinofuranosidase activities (Mewis et al., 2016). This family is divided into 39 subfamilies, and its members act more specifically on arabinoxylan, although its specificity range can be underestimated due to the use of simple synthetic substrates like *p*NP-glycosides in detection. GH43 β-xylosidases were far less numerous than GH3 enzymes in the species studied. Their phylogenetic tree, divided into two major clades, showed overall good bootstrap values (Supplementary Figure S1). The first clade contains enzymes from subfamily 29 with a CBM6 and generally have a signal peptide. It includes all GH43 β-xylosidases from section *Talaromyces*, as well as from two species of *Penicillium* sect. *Lanata-divaricata*. The lower clade groups β-xylosidases from subfamily 1 that lack both CBM and signal peptide and are approximately 50–80 amino acids shorter than those from subfamily 29. These enzymes, which display broader distribution across taxa, appear in the remaining taxonomic groups analyzed, regardless of their habitat and lifestyle. Interestingly, *P. subrubescens* and *P. oxalicum* are soil species with both types of GH43 β-xylosidases.

Overall, GH3 and GH43 families exhibit contrasting diversification patterns. GH3 sequences show broader phylogenetic dispersion, whereas GH43 sequences tend to cluster into more conserved clades. These patterns suggest lineage-associated diversification rather than strict ecological segregation, with closely related species sharing similar repertoires while still displaying genus-level differences.

### 3.4 Phylogenetic analysis of GH10 and GH11 endoxylanases

GH10 enzymes are primarily endo-β-1,4-xylanases that randomly cleave internal β-1,4-xylosidic bonds in the main backbone not only of linear xylan, but also on branched polymers like arabino- and glucuronoxylans, as well as some cellulosic substrates (Gibson et al., 2011; Lyu et al., 2015). Many GH10 endoxylanases have a signal peptide and are modular, with a CBM attached to their catalytic domain. Their active site is located at the C-terminus, in a shallow, wide and open cleft that allows accommodating substitutions on the xylan backbone (Pollet et al., 2010). Therefore, these enzymes are crucial to release xylooligosaccharides hydrolysable by β-xylosidases (Pozo-Rodríguez et al., 2022).

The phylogenetic tree of this group of endoxylanases is very heterogeneous and reflects a high degree of diversification within the species studied (Figure 2), which is in good agreement with their substrate (Pozo-Rodríguez et al., 2022). The upper clade clusters only enzymes from section *Talaromyces* that bear a CBM1. This type of module, that has strong affinity for crystalline cellulose, anchors the catalytic domain to the cellulose surface ensuring its close contact with the substrate. This enables the efficient removal of lignocellulosic xylan, which contributes to making cellulose more accessible for cellulases attack (Arantes and Saddler, 2010). The next clades mostly comprise enzymes from *Penicillium* species formed by a mixture of fungi from the different sections, without a clear aggrupation by taxonomy or habitat. Except for those located in the last clade, which do not have a CBM, the rest of the enzymes also have a CBM1.

In contrast to the high substrate promiscuity of GH10 enzymes, the GH11 β-1,4-endoxylanases are much more specialized. They target only internal β-1,4-xylosidic linkages in xylan and require three unsubstituted consecutive xylose residues to act (Pollet et al., 2010; Paës et al., 2012). Most of them are extracellular enzymes, and some can carry a CBM1. The vast majority of the GH11 endoxylanases identified in this study belonged to sections *Talaromyces* and *Islandici* of the genus *Talaromyces* and *Lanata-di*var*icata* from *Penicillium*. This finding relates the presence of GH11 enzymes with a lifestyle predominantly based on xylan decomposition in plant soils and biomass waste. In our study, only 18 out of 98 endoxylanases have a CBM, but they are not grouped into a specific clade, being scattered throughout the tree.

The phylogenetic tree of these enzymes is complex and profusely branched (Figure 3). At first glance, a closer relatedness between enzymes from section *Talaromyces* and *Penicillium* sect. *Lanata-di*var*icata* is observed, even though the numerous GH11 endoxylanases of these groups appear distributed in different clades and subclades. This pattern is consistent with diversification among taxa occupying different reported ecological contexts.

The tree is divided into two main clades, with the first one featuring only GH11 endoxylanases from section *Talaromyces* and *Penicillium* sect. *Lanata-divaricata*. The upper branches of the second clade have also many enzymes from species belonging to these two sections, while the few GH11 endoxylanases produced by species associated with other environmental conditions are completely separated and grouped in the lowest subclades of the tree. None of those, related to fruit, dry habitats, and pathogenic fungi, possess CBMs, suggesting independent diversification across lineages, or to contribute to cell wall attack during infection of healthy plants. This specialization represents an opportunity to study these enzymes further to uncover new functionalities and applications. Analyzing the differences between enzymes in the various branches can provide insights into their evolutionary paths and sequence features, helping to understand why they have evolved distinct characteristics and functionalities.

### 3.5 Phylogenetic analysis of GH51, GH54 and GH62 *α*-L-arabinofuranosidases

In plants, arabinose is predominantly present as α-L-arabinofuranose, forming part of arabinoxylans and glucuronoarabinoxylans. The structural diversity of these polysaccharides explains the high variety of enzymes necessary to degrade them and the huge amount α-L-arabinofuranosidases identified in this study. In CAZy, they are mostly grouped in families GH51, GH54 and GH62.

GH51 enzymes primarily exhibit arabinofuranosidase activity (Yang et al., 2015), releasing (1 → 2) and (1 → 3)-α-L-arabinofuranosyl residues from mono-substituted xylopyranosyl residues in arabinoxylan (Poria et al., 2020). Phylogenetic analysis of the 99 GH51 candidates found in this study showed two well-delimited clades (Supplementary Figure S2), both split into two branches, suggesting different evolutionary trajectories. The first clade is highly heterogeneous in terms of fungal lifestyle, especially its lower subclade, that is larger and includes species from nearly all sections studied in this work. Enzymes classified in GH51 subfamily 2 appear in both main clades, while subfamily 1 arabinofuranosidases, that lack a signal peptide, cluster as a homogeneous subclade in the second one, separated from the rest by a large evolutionary distance. Fungi in this subclade range from saprophytic to parasitic lifestyles, and the putative involvement of these enzymes in intracellular processes makes this group interesting.

On the other hand, most GH54 enzymes are extracellular fungal arabinofuranosidases with a modular structure, possessing a CBM42 at their C-terminus that aids in binding to arabinoxylan (Miyanaga et al., 2006). About 75% of the GH54 arabinofuranosidases identified in this study originate from species of *Talaromyces* and *Penicillium* sect. *Lanata-divaricata*, typical inhabitants of soils. Their phylogenetic tree (Supplementary Figure S3) mirrors the pattern seen in GH11, revealing their evolutionary conservation.

Finally, GH62 enzymes are specialized debranching arabinofuranosidases active on arabinoxylan. The GH62 candidates are extracellular enzymes, with a catalytic domain of approximately 265–275 amino acids. Their phylogenetic analysis (Supplementary Figure S4) revealed two well-supported clades, with clear evolutionary patterns and potentially reflecting functional divergence. The first clade predominantly clusters *Talaromyces* enzymes, with only four members from *Penicillium* sect. *Lanata-divaricata*. The second clade contains only modular enzymes with a CBM1 in the N-terminal region, produced by *Penicillium* species. Despite the structural similarity of the catalytic domain of these enzymes to those of *Talaromyces*, the phylogenetic distance between these groups suggests a possible divergence in their evolutionary paths. Remarkably, several species from section *Lanata-divaricata* can be found in both clades, suggesting a high sequence diversity within this fungal group.

### 3.6 Phylogenetic analysis of α-glucuronidases (GH67)

Glucuronidases from GH67 family target glucuronic acid or 4-*O*-methyl-D-glucuronic acid side chains attached by α-1,2 linkage to the xylan backbone (Malgas et al., 2021). In filamentous fungi, they are predominantly secreted enzymes, consistent with their role in extracellular degradation of glucuronoxylans. The topology of the phylogenetic tree of the GH67 α-glucuronidases identified in the current investigation (Supplementary Figure S5) is similar to those of GH3, GH11, GH43 and GH62 enzymes, with a main clade including species (from sections *Talaromyces* and *Lanata-divaricata*), and a second one that gathers enzymes from all studied taxa. Interestingly, GH67 enzymes are more represented in taxa related to plant biomass residues and soil-derived substrates than in species from other habitats (Supplementary Table SIII). It should also be noticed that the enzymes in both clades have similar size and a signal peptide. The low number of characterized α-glucuronidases (only 27 according to CAZy, only 6 of them eukaryotic) makes it challenging to determine the exact complexity of this family.

### 3.7 Phylogenetic analysis of carbohydrate esterases (CE1 and CE5)

The CE1 family in the CAZy database includes fungal acetylxylan esterases (AXEs) and feruloyl esterases (FAEs) (Oliveira et al., 2019; Li et al., 2020). CE1 enzymes are recognized for their role in xylan degradation and the production of bioactive compounds. Specifically, acetylxylan esterases catalyze the hydrolysis of ester linkages between a xylopyranose residue and its acetyl substituent, while feruloyl esterases hydrolyze ferulic acid residues esterified to certain arabinoses in the side chains of xylan.

CE1 enzymes are scarcely represented in the species analyzed and restricted to *Talaromyces* and *Penicillium* sect. *Lanata-divaricata* (Supplementary Table SIII), that share the same type of habitats (plant biomass-rich environments) and lifestyle. All feruloyl esterases identified are from *Talaromyces* species and appear scattered in different branches at the bottom of the tree (Supplementary Figure S6), well separated from acetylxylan esterases, showcasing distinct evolutionary pathways and enzyme activities. Notably, CE1 feruloyl esterases feature a CBM1 at their C-terminal end and have a signal peptide.

CE5 esterases are involved in the deacetylation of polysaccharides. However, some CE5 enzymes have recently been identified as cutinases capable of degrading some plastics like polyethylene terephthalate (Novy et al., 2021). The production of CE5 enzymes is particularly valuable in industrial applications where the deacetylation of biomass is necessary to produce biofuels and other value-added products.

Analysis of the CE5 phylogeny allowed observing distinct clades (Supplementary Figure S7), indicating different evolutionary trajectories. The enzymes are well-clustered according to the lifestyle of the fungal species. Two sections of *Talaromyces* specialized in plant biomass degradation, whose CE5 esterases bear a CBM1, are prominent. The middle-lower part of the tree groups enzymes from fruit and plant pathogens, particularly citrus and apples. Given the high cutin content in these fruits, it is likely that these clades include enzymes that, in addition to their deacetylation role, are active on cutin.

Taken together, the different CAZy families analyzed reveal a recurrent pattern: closely related species tend to share similar repertoires, while broader ecological categories encompass heterogeneous enzymatic profiles. This indicates that phylogenetic history represents a primary structuring factor, whereas ecological associations appear as contextual rather than deterministic.

### 3.8 Taxonomy and eco-physiological groups

The phylogenetic patterns observed across CAZy families revealed several evolutionary trends. Below, we discuss these patterns, highlighting how lifestyle in *Penicillium* is reflected in more heterogeneous enzymatic repertoires, whereas *Talaromyces* shows a more uniform distribution consistent with its comparatively narrower ecological range.

**Figure 4:**
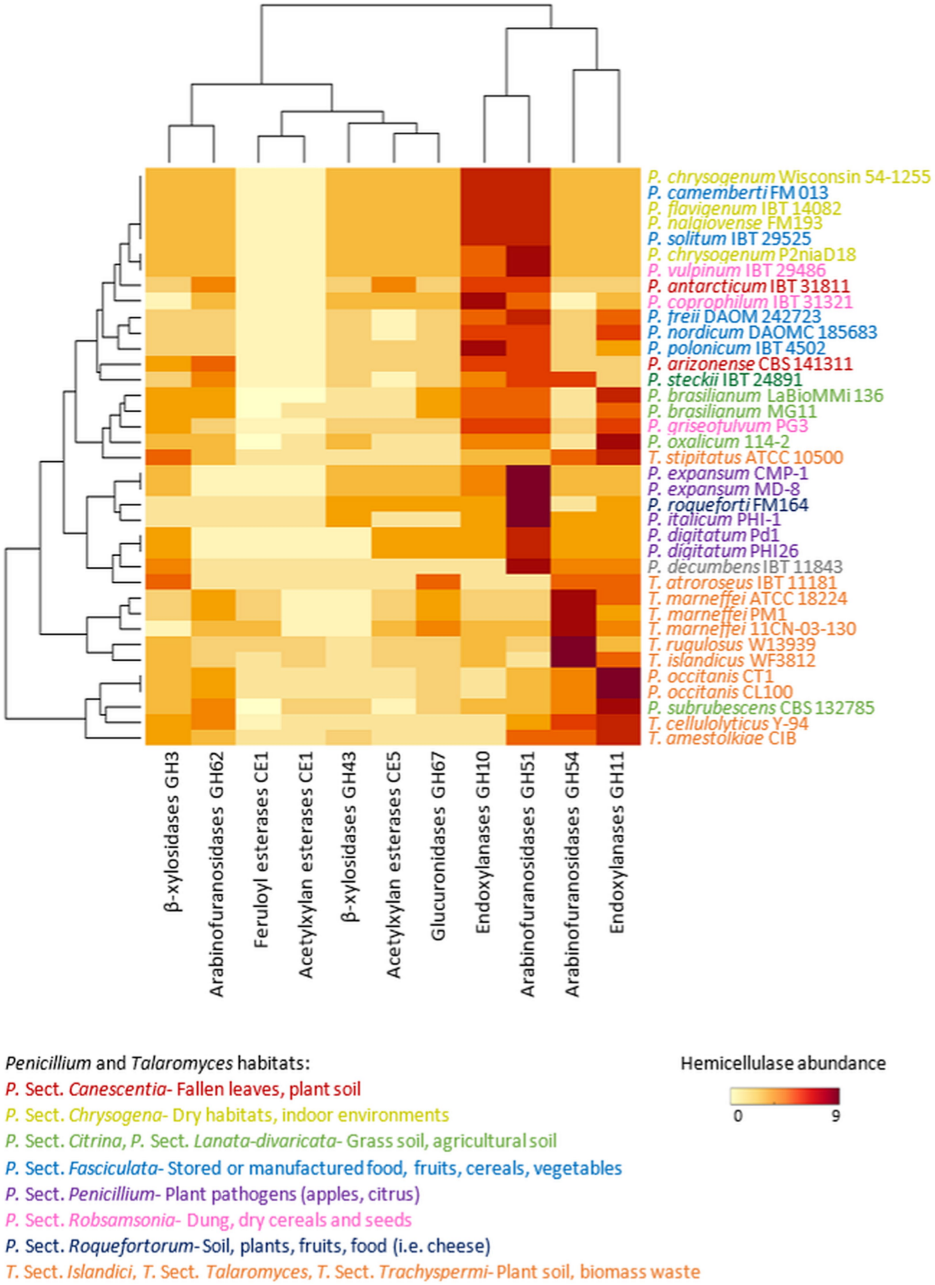
Hierarchical clustering and heatmap of xylan-active hemicellulase categories across the 37 *Penicillium* and *Talaromyces* genomes. Species (rows) and enzyme categories (columns) were grouped by hierarchical clustering (Euclidean distance, complete linkage). Colour intensity reflects the number of sequences per category (0–9; see scale). Note that CE1 is shown as two functional categories (acetylxylan and feruloyl esterases), giving 11 categories from 10 CAZy families. Species names are colour-coded by taxonomic section according to their reported habitat (see key).

To visualize all these features simultaneously, we performed a multivariate clustering analysis of the fungal species based on their xylanolytic enzyme repertoire. The enzyme categories included in the study were: β-xylosidases (GH3, GH43), endoxylanases (GH10, GH11), arabinofuranosidases (GH51, GH54, GH62), glucuronidases (GH67), acetylxylan esterases (CE1, CE5), and feruloyl esterases (CE1). The results are displayed in a heatmap (Figure 4), which uses hierarchical clustering to relate the *Penicillium* and *Talaromyces* species (in rows), with specific enzyme families (in columns).

Clustering disclosed patterns of enzyme distribution across ecological categories, highlighting marked differences in enzyme activities between pathogenic and saprophytic species. Two different distribution patterns can be observed among the saprophytes studied. Most of them (in the upper half of Figure 4) display a similar pattern, with high representation of sequences that codify endoxylanases of the versatile GH10 family and GH51 arabinofuranosidases. This correlation reflects the functional synergy of both activities in xylan degradation, as well as the selection of this combination across evolution in this group of species that, in general, have less genes corresponding to other xylanolytic categories. A distinct pattern was observed for species belonging to section *Talaromyces* (bottom of Figure 4), which display comparatively fewer GH10 and GH51 sequences but a higher representation of GH11 and GH54 families. These enzymes form consistent phylogenetic groupings within the section, suggesting lineage-associated repertoire profiles. Rather than indicating specific adaptive strategies, this pattern reflects differential distribution of CAZy families among closely related taxa. Species associated with soil and decomposing substrates therefore show characteristic enzyme complements without implying functional specialization.

In turn, plant pathogenic species have also a different pattern, with the highest proportion of genes that encode GH51 arabinofuranosidases and fewer sequences encoding GH10 and GH11 enzymes (equally represented). The lifestyle of these microorganisms probably shows a broader repertoire of predicted CAZymes than act over living and intact plant tissues.

This analysis also showed a correlation between CE5 acetylxylan esterases and GH 67 glucuronidases, which may reflect a coadaptation to overcome chemical substitutions in xylans. Finally, the genes of GH3 xylosidases and GH62 arabinofuranosidases seem to be evolutionarily linked among saprophytes, although GH62 genes are poorly represented in the plant pathogens studied. GH3 enzymes are not only pivotal for utilizing complex polysaccharides of decaying plant material in saprophytes, but also for penetrating plant defenses in pathogens, which can explain their wide distribution regardless of fungal lifestyle.

From an applied perspective, phylogenomic analyses like the one presented here can help prioritize candidates for future biochemical characterization. Mapping uncharacterized sequences onto well-supported clades that contain experimentally validated hemicellulases enables the identification of promising candidates for subsequent biochemical testing. In practice, candidates prioritized through this framework would be heterologously expressed (e.g., in *Pichia pastoris* or *Aspergillus* hosts), purified, and assayed against defined substrates, such as beechwood or wheat arabinoxylan and xylo-oligosaccharides, to confirm their activity and substrate specificity, followed by biophysical characterization of their thermostability, pH optima and kinetic parameters. Enzymes displaying favourable properties could then be incorporated into applications such as lignocellulosic biomass saccharification, prebiotic xylo-oligosaccharide production, animal feed, and the pulp and paper industry (Kuhad et al., 2011; Shrivastava, 2020). Indeed, several of the enzyme types surveyed here have already been functionally characterized, and even engineered, in one of the species included in this study: the *β*-xylosidase and endoxylanase activities of *Talaromyces amestolkiae* were recently enhanced through genetic manipulation of the transcriptional activator XlnR (Pozo-Rodríguez et al., 2025), illustrating how candidates identified within this evolutionary framework can be translated into experimentally validated and improved biocatalysts. In addition, recognizing lineage-specific expansions in families such as GH10, GH11, GH43 or GH62 can help prioritize species or strains with enriched xylanolytic potential for biotechnological screening. While the present study does not evaluate enzymatic activities experimentally, the evolutionary framework established here provides a rational basis for selecting promising targets for future functional and biotechnological investigations.

Several limitations should be considered when interpreting these results. First, the analysis relies on publicly available, computationally predicted proteomes of uneven assembly and annotation quality, and on homology-based functional assignment. Because CAZy families such as GH43 and CE1 are functionally heterogeneous, the activities reported here are predicted rather than experimentally validated. Second, the ecological categories were compiled from literature descriptions and used only as qualitative comparative metadata, without quantitative or statistical testing, so the associations between enzyme repertoires and lifestyle should be regarded as hypotheses for future validation. Third, taxon sampling is uneven across sections and genera, and the phylogenetic reconstructions are based on conserved catalytic domains, so the resulting topologies reflect family-level sequence relationships rather than species phylogenies.

## 4 Conclusion

This study provides a comparative phylogenomic overview of xylan-active CAZyme repertoires in *Talaromyces* and *Penicillium*. Because the reconstructions are based on the conserved catalytic domains of each family, the resulting topologies primarily reflect family-level sequence relationships; within this framework, gene distribution appears to be structured mainly by taxonomic relatedness, with lineage-associated expansions and conserved clades that co-occur with particular ecological categories. Overall, these results establish a curated evolutionary framework that facilitates the interpretation of CAZyme diversity in these genera and, by mapping uncharacterized sequences onto clades containing experimentally validated enzymes, provides a rational basis for prioritizing candidates for future biochemical characterization and biotechnological exploration.

## Data Availability

The original contributions presented in the study are included in the article/Supplementary material, further inquiries can be directed to the corresponding author.

## Footnotes

1
https://mycocosm.jgi.doe.gov

2
https://www.uniprot.org/

3
https://mafft.cbrc.jp/alignment/server/

4
http://tree.bio.ed.ac.uk/software/figtree

5
http://www.rstudio.com
