# Supplementary material for: Phylogenetic analysis of xylan-degrading hemicellulases from Talaromyces and Penicillium: taxonomic and ecophysiological implications
Source: Front Microbiol. 2026 Jul 15;17:1818172. doi: 10.3389/fmicb.2026.1818172 (PMC13415672; doi:10.3389/fmicb.2026.1818172)
Supplement: Supplementary file 1 [file Table_1.DOCX]

**Table SI. *Penicillium* and *Talaromyces* proteomes retrieved from JGI and Uniprot for this study.**

|  | **Section** | **##** | **Fungus** | **Proteins** | **Publication** |
| --- | --- | --- | --- | --- | --- |
| Mycocosom (JGI) | P. sect. *Canescentia* | 1 | [*Penicillium antarcticum* IBT 31811](https://mycocosm.jgi.doe.gov/Penant1) (Pant) | 11,514 | [Nielsen JC et al., 2017](https://www.ncbi.nlm.nih.gov/pubmed/28368369) |
|  |  | 2 | [*Penicillium arizonense* CBS 141311](https://mycocosm.jgi.doe.gov/Penar1) (Pari) | 12,253 | [Grijseels S et al., 2016](https://www.ncbi.nlm.nih.gov/pubmed/27739446) |
|  | P. sect. *Chrysogena* | 3 | [*Penicillium chrysogenum* Wisconsin 54-1255](https://mycocosm.jgi.doe.gov/PenchWisc1_1) (Pchry_Wisconsin54-1255) | 13,671 | [van den Berg MA et al., 2008](https://www.ncbi.nlm.nih.gov/pubmed/18820685) |
|  |  | 4 | [*Penicillium flavigenum* IBT 14082](https://mycocosm.jgi.doe.gov/Penfla1) (Pfla) | 10,994 | [Nielsen JC et al., 2017](https://www.ncbi.nlm.nih.gov/pubmed/28368369) |
|  |  | 5 | [*Penicillium nalgiovense* FM193](https://mycocosm.jgi.doe.gov/Pennal1) (Pnal) | 11,853 | [Nielsen JC et al., 2017](https://www.ncbi.nlm.nih.gov/pubmed/28368369) |
|  | P. sect. *Citrina* | 6 | [*Penicillium steckii IBT* 24891](https://mycocosm.jgi.doe.gov/Penste1) (Pste) | 10,362 | [Nielsen JC et al., 2017](https://www.ncbi.nlm.nih.gov/pubmed/28368369) |
|  | P. sect. *Exilicoulis* | 7 | [*Penicillium decumbens* IBT 11843](https://mycocosm.jgi.doe.gov/Pendec1) (Pde) | 7,149 | [Nielsen JC et al., 2017](https://www.ncbi.nlm.nih.gov/pubmed/28368369) |
|  | P. sect. *Fasciculata* | 8 | [*Penicillium camemberti* FM 013](https://mycocosm.jgi.doe.gov/Pencam1) (Pcam) | 14,418 | [Cheeseman K et al., 2014](https://www.ncbi.nlm.nih.gov/pubmed/24407037) |
|  |  | 9 | [*Penicillium freii* DAOM 242723](https://mycocosm.jgi.doe.gov/Penfr1) (Pfr) | 11,739 | [Wingfield BD et al., 2016](https://www.ncbi.nlm.nih.gov/pubmed/27433447) |
|  |  | 10 | [*Penicillium nordicum* DAOMC 185683](https://mycocosm.jgi.doe.gov/Pennord1) (Pnord) | 12,959 | [Wingfield BD et al., 2015](https://www.ncbi.nlm.nih.gov/pubmed/26734552) |
|  |  | 11 | [*Penicillium polonicum* IBT 4502](https://mycocosm.jgi.doe.gov/Penpol1) (Ppol) | 10,694 | [Nielsen JC et al., 2017](https://www.ncbi.nlm.nih.gov/pubmed/28368369) |
|  |  | 12 | [*Penicillium solitum* IBT 29525](https://mycocosm.jgi.doe.gov/Pensol1) (Psol) | 11,889 | [Nielsen JC et al., 2017](https://www.ncbi.nlm.nih.gov/pubmed/28368369) |
|  | P. sect. *Lanata-divaricata* | 13 | [*Penicillium brasilianum* MG11](https://mycocosm.jgi.doe.gov/Penbra1) (Pbra_MG11) | 11,943 | [Horn F et al., 2015](https://www.ncbi.nlm.nih.gov/pubmed/26337871) |
|  |  | 14 | [*Penicillium oxalicum* 114-2](https://mycocosm.jgi.doe.gov/Penox1) (Pox) | 9,979 | [Liu G et al., 2013](https://www.ncbi.nlm.nih.gov/pubmed/23383313) |
|  |  | 15 | [*Penicillium subrubescens* CBS 132785](https://mycocosm.jgi.doe.gov/Pensub1) (Psub) | 14,188 | [Peng M et al., 2017](https://www.ncbi.nlm.nih.gov/pubmed/28216099) |
|  | P. sect. *Penicillium* | 16 | [*Penicillium digitatum* PHI26](https://mycocosm.jgi.doe.gov/Pendi1) (Pdi_PHI26) | 9,118 | [Marcet-Houben M et al., 2012](https://www.ncbi.nlm.nih.gov/pubmed/23171342) |
|  |  | 17 | [*Penicillium digitatum* Pd1](https://mycocosm.jgi.doe.gov/Pendig1) (Pdi_Pd1) | 8,946 | [Marcet-Houben M et al., 2012](https://www.ncbi.nlm.nih.gov/pubmed/23171342) |
|  |  | 18 | [*Penicillium italicum* PHI-1](https://mycocosm.jgi.doe.gov/Penita1) (Pita) | 9,996 | [Ballester AR et al., 2015](https://www.ncbi.nlm.nih.gov/pubmed/25338147) |
|  | P. sect. *Robsamsonia* | 19 | [*Penicillium coprophilum* IBT 31321](https://mycocosm.jgi.doe.gov/Pencop1) (Pcop) | 8,999 | [Nielsen JC et al., 2017](https://www.ncbi.nlm.nih.gov/pubmed/28368369) |
|  |  | 20 | [*Penicillium griseofulvum* PG3](https://mycocosm.jgi.doe.gov/Pengri1) (Pgri) | 9,63 | [Banani H et al., 2016](https://www.ncbi.nlm.nih.gov/pubmed/26729047) |
|  |  | 21 | [*Penicillium vulpinum* IBT 29486](https://mycocosm.jgi.doe.gov/Penvul1) (Pvul) | 10,278 | [Nielsen JC et al., 2017](https://www.ncbi.nlm.nih.gov/pubmed/28368369) |
|  | P. sect. *Roquefortorum* | 22 | [*Penicillium roqueforti* FM164](https://mycocosm.jgi.doe.gov/Penro1) (Pro) | 12,319 | [Cheeseman K et al., 2014](https://www.ncbi.nlm.nih.gov/pubmed/24407037) |
|  | T. sect. *Islandici* | 2 | [*Talaromyces islandicus* WF3812](https://mycocosm.jgi.doe.gov/Talis1) (Tis) | 9,927 | [Schafhauser T et al., 2015](https://www.ncbi.nlm.nih.gov/pubmed/26197417) |
|  | T. sect. *Talaromyces* | 24 | [*Penicillium occitanis* CL100](https://mycocosm.jgi.doe.gov/Penoc1) (Poc_CL100) | 11,231 | [Bravo-Ruiz G et al., 2017](https://www.ncbi.nlm.nih.gov/pubmed/28951729) |
|  |  | 25 | [*Talaromyces marneffei* ATCC 18224](https://mycocosm.jgi.doe.gov/Talma1_2) (Tma_ATCC18224) | 10,638 | [Nierman WC et al., 2015](https://pubmed.ncbi.nlm.nih.gov/25676766/) |
|  |  | 26 | [*Talaromyces stipitatus* ATCC 10500](https://mycocosm.jgi.doe.gov/Talst1_2) (Tst) | 13,252 | [Nierman WC et al., 2015](https://pubmed.ncbi.nlm.nih.gov/25676766/) |
| Uniprot | P. sect. *Chrysogena* | 27 | [*Penicillium chrysogenum* P2niaD18](https://www.uniprot.org/proteomes/UP000076449) (Pchry_P2niaD18) | 11,144 | [Specht T et al., 2014](https://pubmed.ncbi.nlm.nih.gov/25059858/) |
|  | P. sect. *Lanata-divaricata* | 28 | [*Penicillium brasilianum* LaBioMMi 136](https://www.uniprot.org/proteomes/UP000190744) (Pbra_LaBioMMi 136) | 10,352 | [Fill TP et al., 2018](https://pubmed.ncbi.nlm.nih.gov/30533840/) |
|  | P. sect. *Penicillium* | 29 | [*Penicillium expansum* CMP-1](https://www.uniprot.org/proteomes/UP000030155) (Pex_CMP1) | 10,663 | [Ballester AR et al., 2015](https://pubmed.ncbi.nlm.nih.gov/25338147/) |
|  |  | 30 | [*Penicillium expansum* MD-8](https://www.uniprot.org/proteomes/UP000030143) (Pex_MD8) | 11,06 | [Ballester AR et al., 2015](https://pubmed.ncbi.nlm.nih.gov/25338147/) |
|  | T. sect. *Islandici* | 31 | [*Talaromyces rugulosus* W13939](https://www.uniprot.org/proteomes/UP000509510) (Tru) | 11,83 | [Wang B et al., 2020](https://pubmed.ncbi.nlm.nih.gov/33048598/) |
|  | T. sect. *Talaromyces* | 32 | [*Penicillium occitanis* CT1](https://www.uniprot.org/proteomes/UP000217506) (Poc_CT1) | 11,267 | [Bravo-Ruiz G et al., 2017](https://pubmed.ncbi.nlm.nih.gov/28951729/) |
|  |  | 33 | [*Talaromyces amestolkiae* CIB](https://www.uniprot.org/proteomes/UP000249363) (Tam) | 10,407 | [de Eugenio LI et al., 2017](https://pubmed.ncbi.nlm.nih.gov/28649280/) |
|  |  | 34 | [*Talaromyces celluloyticus* Y-94](https://www.uniprot.org/proteomes/UP000053095) (Tce) | 10,907 | [Fujii T et al., 2015](https://pubmed.ncbi.nlm.nih.gov/25720677/) |
|  |  | 35 | [*Talaromyces marneffei* PM1](https://www.uniprot.org/proteomes/UP000029285) (Tma_PM1) | 12,153 | [Yang E et al., 2014](https://pubmed.ncbi.nlm.nih.gov/25330172/) |
|  |  | 36 | [*Talaromyces marneffei* 11CN-03-130](https://www.uniprot.org/proteomes/UP000480676) (Tma_11CN-03-130) | 10,013 | [Cuomo CA et al., 2020](https://pubmed.ncbi.nlm.nih.gov/31919177/) |
|  | T. sect. *Trachyspermi* | 37 | *Talaromyces atroroseus* IBT 11181 (Tat) | 9,520 | Thrane U et al. 2017 |

|  | **EC number** | **CAZy family** | **Characterized sequences for BLASTp** | |
| --- | --- | --- | --- | --- |
| **Hemicellulases groups active on xylan** | Endoxylanases  EC 3.2.1.8 | GH10 | [Q6PRW6](https://www.uniprot.org/uniprot/Q6PRW6)  *P. chrysogenum* FS010 | [Q9P8J1](https://www.uniprot.org/uniprot/Q9P8J1)  *T. purpureogenus* MYA-38 |
|  |  | GH11 | [E7EF85](https://www.uniprot.org/uniprot/E7EF85)  *P. oxalicum* B3-11-2 | [Q96W72](https://www.uniprot.org/uniprot/Q96W72)  *T. purpureogenus* MYA-38 |
|  | β-xylosidases  EC 3.2.1.37 | GH3 | [G3XFU3](https://www.uniprot.org/uniprot/G3XFU3)  *T. cellulolyticus* CF2612 | [A0A0F7KMN1](https://www.uniprot.org/uniprot/A0A0F7KMN1)  *T. purpureogenus* MYA-38 |
|  |  | GH43 | [EPS31351.1](https://www.ncbi.nlm.nih.gov/protein/EPS31351.1/)  *P. oxalicum* 1142 | [Q870E8](https://www.uniprot.org/uniprot/Q870E8)  *P. herquei* |
|  | α-L-arabinofuranosidases  EC 3.2.1.55 | GH51 | [B5MGR0](https://www.uniprot.org/uniprot/B5MGR0)  *P. chrysogenum* 31B | [A4UU45](https://www.uniprot.org/uniprot/A4UU45)  *T. purpureogenus* MYA-38 |
|  |  | GH54 | [B5MGR1](https://www.uniprot.org/uniprot/B5MGR1)  *P. chrysogenum* 31B | [Q96X02](https://www.uniprot.org/uniprot/Q96X02)  *T. purpureogenus* MYA-38 |
|  |  | GH62 | [B5MGR2](https://www.uniprot.org/uniprot/B5MGR2)  *P. chrysogenum* 31B | [A0A2H5BN17](https://www.uniprot.org/uniprot/A0A2H5BN17)  *T. pinophilus t179-11* |
|  | α-glucuronidases  EC 3.2.1.139 | GH67 | [Q5AQZ4](https://www.uniprot.org/uniprot/Q5AQZ4)  *A. nidulans* FGSC A4 | [Q8X211](https://www.uniprot.org/uniprot/Q8X211)  *R. emersonii* CBS 814.70 |
|  | Acetylxylan esterases  EC 3.1.1.72 | CE1 | [Q75P26](https://www.uniprot.org/uniprot/Q75P26)  *A. oryzae* RIB40 | [Q8NJP6](https://www.uniprot.org/uniprot/Q8NJP6)  *T. purpureogenus* MYA-38 |
|  |  | CE5 | [CAZ65690.1](https://www.ncbi.nlm.nih.gov/protein/CAZ65690.1)  *P. aurantiogriseum* | [O59893](https://www.uniprot.org/uniprot/O59893)  *T. purpureogenus* MYA-38 |
|  | Feruloyl esterases  EC 3.1.1.73 | CE1 | [Q9HE18](https://www.uniprot.org/uniprot/Q9HE18)  *T. funiculosus* | [B8M9H9](https://www.uniprot.org/uniprot/B8M9H9)  *T. stipitatus* |

**Table SII. Query sequences used in BLASTp to retrieve the hemicellulases active on xylan that are present in *Penicillium* and *Talaromyces* theoretical proteomes.**

**Table SIII. Fungal species and number of sequences in each family of hemicellulases active on xylan.**

|  |  | **Endoxylanases** | | **β-xylosidases** | | **α-L-arabinofuranosidases** | | | **α-glucuronidases** | **Acetyl xylan esterases** | | **Feruloyl esterases** |
| --- | --- | --- | --- | --- | --- | --- | --- | --- | --- | --- | --- | --- |
|  |  | **GH10** | **GH11** | **GH3** | **GH43** | **GH51** | **GH54** | **GH62** | **GH67** | **CE1** | **CE5** | **CE1** |
| ***Penicillium*** | | | | | | | | | | | | |
| Sect. *Canescentia* | Pant | 3 | 1 | 1 | 1 | 3 | 1 | 2 | 1 | 0 | 2 | 0 |
|  | Pari | 4 | 1 | 2 | 1 | 4 | 1 | 3 | 1 | 0 | 1 | 0 |
| Sect. *Chrysogena* | Pchry_Wisconsin54-1255 | 3 | 1 | 1 | 1 | 3 | 1 | 1 | 1 | 0 | 1 | 0 |
|  | Pch_P2niaD18 | 2 | 1 | 1 | 1 | 3 | 1 | 1 | 1 | 0 | 1 | 0 |
|  | Pfla | 3 | 1 | 1 | 1 | 3 | 1 | 1 | 1 | 0 | 1 | 0 |
|  | Pnal | 3 | 1 | 1 | 1 | 3 | 1 | 1 | 1 | 0 | 1 | 0 |
| Sect. *Citrina* | Pste | 2 | 1 | 1 | 1 | 3 | 3 | 2 | 1 | 0 | 0 | 0 |
| Sect. *Exilicoulis* | Pde | 0 | 1 | 1 | 0 | 2 | 1 | 0 | 0 | 0 | 0 | 0 |
| Sect. *Fasciculata* | Pcam | 3 | 1 | 1 | 1 | 3 | 1 | 1 | 1 | 0 | 1 | 0 |
|  | Pfr | 3 | 3 | 1 | 1 | 4 | 1 | 1 | 1 | 0 | 0 | 0 |
|  | Pnord | 3 | 3 | 1 | 1 | 3 | 1 | 1 | 1 | 0 | 0 | 0 |
|  | Ppol | 4 | 2 | 1 | 1 | 3 | 1 | 1 | 1 | 0 | 1 | 0 |
|  | Psol | 3 | 1 | 1 | 1 | 3 | 1 | 1 | 1 | 0 | 1 | 0 |
| Sect. *Lanata-divaricata* | Pbra_MG11 | 3 | 3 | 2 | 1 | 3 | 1 | 2 | 2 | 1 | 1 | 0 |
|  | Pbra_LaBioMMi136 | 3 | 4 | 2 | 1 | 3 | 1 | 2 | 2 | 0 | 1 | 0 |
|  | Pox | 3 | 5 | 2 | 2 | 3 | 1 | 2 | 1 | 1 | 1 | 0 |
|  | Psub | 3 | 7 | 3 | 2 | 3 | 4 | 4 | 2 | 2 | 1 | 0 |
| Sect. *Penicillium* | Pdi_PHI26 | 1 | 1 | 1 | 0 | 2 | 1 | 0 | 1 | 0 | 1 | 0 |
|  | Pdi_Pd1 | 1 | 1 | 1 | 0 | 2 | 1 | 0 | 1 | 0 | 1 | 0 |
|  | Pita | 1 | 1 | 0 | 1 | 3 | 1 | 0 | 0 | 0 | 0 | 0 |
|  | Pex_CMP1 | 2 | 1 | 1 | 1 | 4 | 1 | 0 | 1 | 0 | 1 | 0 |
|  | Pex_MD8 | 2 | 1 | 1 | 1 | 4 | 1 | 0 | 1 | 0 | 1 | 0 |
| Sect. *Robsamsonia* | Pcop | 3 | 1 | 0 | 1 | 2 | 0 | 1 | 1 | 0 | 1 | 0 |
|  | Pgri | 3 | 3 | 2 | 1 | 3 | 1 | 1 | 1 | 0 | 1 | 0 |
|  | Pvul | 2 | 1 | 1 | 1 | 3 | 1 | 1 | 1 | 0 | 1 | 0 |
| Sect. *Roquefortorum* | Pro | 1 | 1 | 0 | 1 | 3 | 0 | 0 | 1 | 0 | 1 | 0 |
| ***Talaromyces*** | | | | | | | | | | | | |
| Sect. *Islandici* | Tis | 2 | 4 | 2 | 0 | 0 | 7 | 1 | 1 | 1 | 1 | 0 |
|  | Tru | 2 | 2 | 2 | 0 | 1 | 7 | 1 | 1 | 1 | 1 | 1 |
| Sect. *Talaromyces* | Tst | 2 | 4 | 3 | 1 | 2 | 3 | 2 | 1 | 1 | 1 | 1 |
|  | Tam | 1 | 8 | 4 | 2 | 6 | 6 | 3 | 1 | 1 | 1 | 1 |
|  | Tce | 1 | 6 | 3 | 1 | 3 | 5 | 4 | 1 | 1 | 1 | 0 |
|  | Tma_ATCC18224 | 1 | 3 | 1 | 0 | 1 | 4 | 2 | 2 | 0 | 1 | 1 |
|  | Tma_PM1 | 1 | 2 | 1 | 0 | 1 | 4 | 2 | 2 | 0 | 1 | 1 |
|  | Tma_11CN-03-130 | 1 | 2 | 0 | 0 | 1 | 4 | 1 | 2 | 0 | 1 | 1 |
| Sect. *Trachyspermi* | Tat | 0 | 1 | 1 | 0 | 0 | 1 | 0 | 1 | 0 | 0 | 0 |
| - | Poc_CL100 | 1 | 9 | 3 | 1 | 3 | 5 | 4 | 2 | 1 | 1 | 1 |
| - | Poc_CT1 | 1 | 9 | 3 | 1 | 3 | 5 | 4 | 2 | 1 | 1 | 1 |
|  |  |  |  |  |  |  |  |  |  |  |  |  |
| **Number of hemicellulases active on xylan** | | 77 | 98 | 53 | 31 | 99 | 80 | 53 | 43 | 11 | 32 | 8 |
| **Total** | | 585 | | | | | | | | | | |


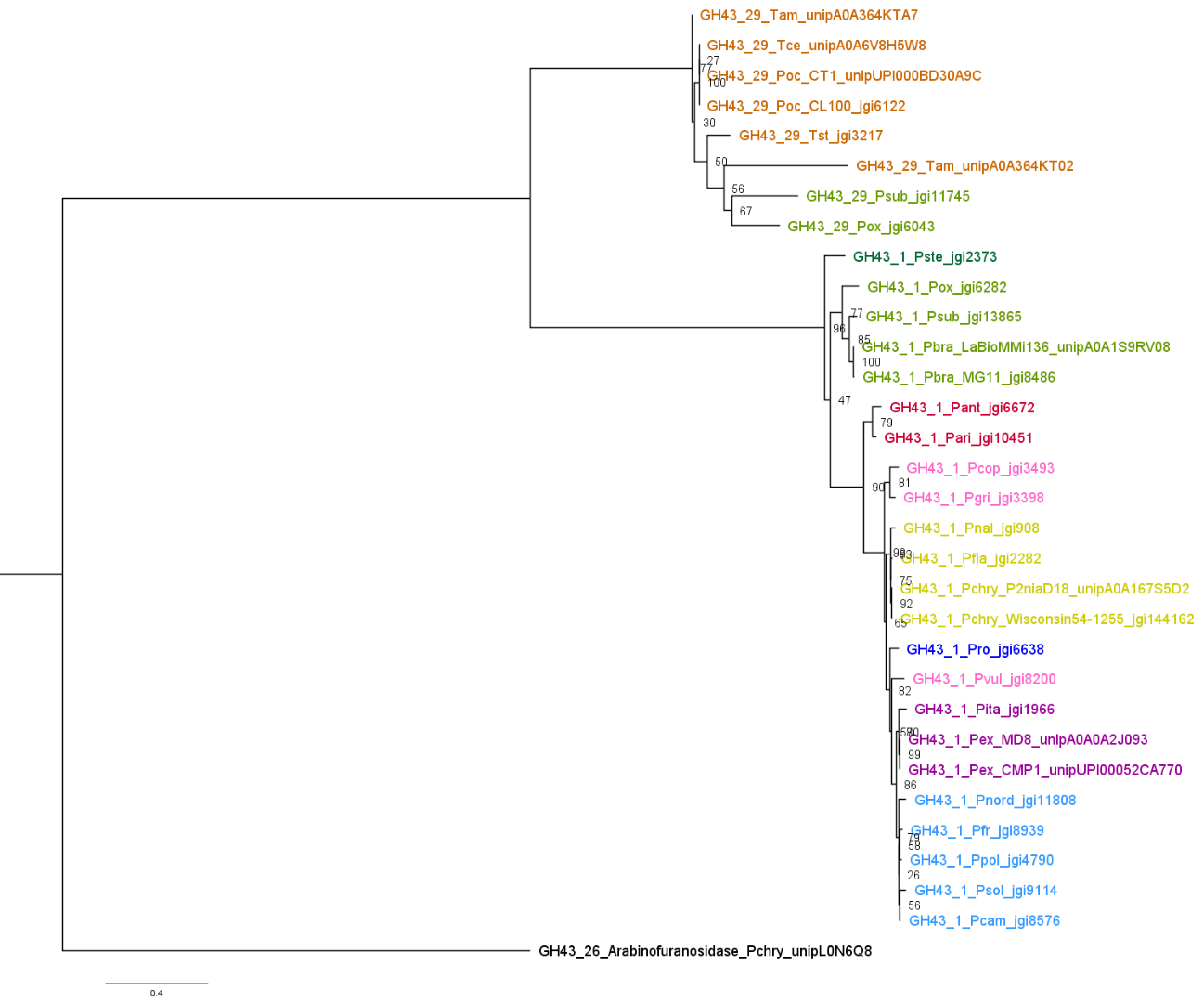


**Figure S1.** Phylogenetic tree of GH43 β-xylosidases from *Penicillium* and *Talaromyces*. The root sequence is a GH43 arabinofuranosidase of subfamily 26 from *P. chrysogenum* 31B.


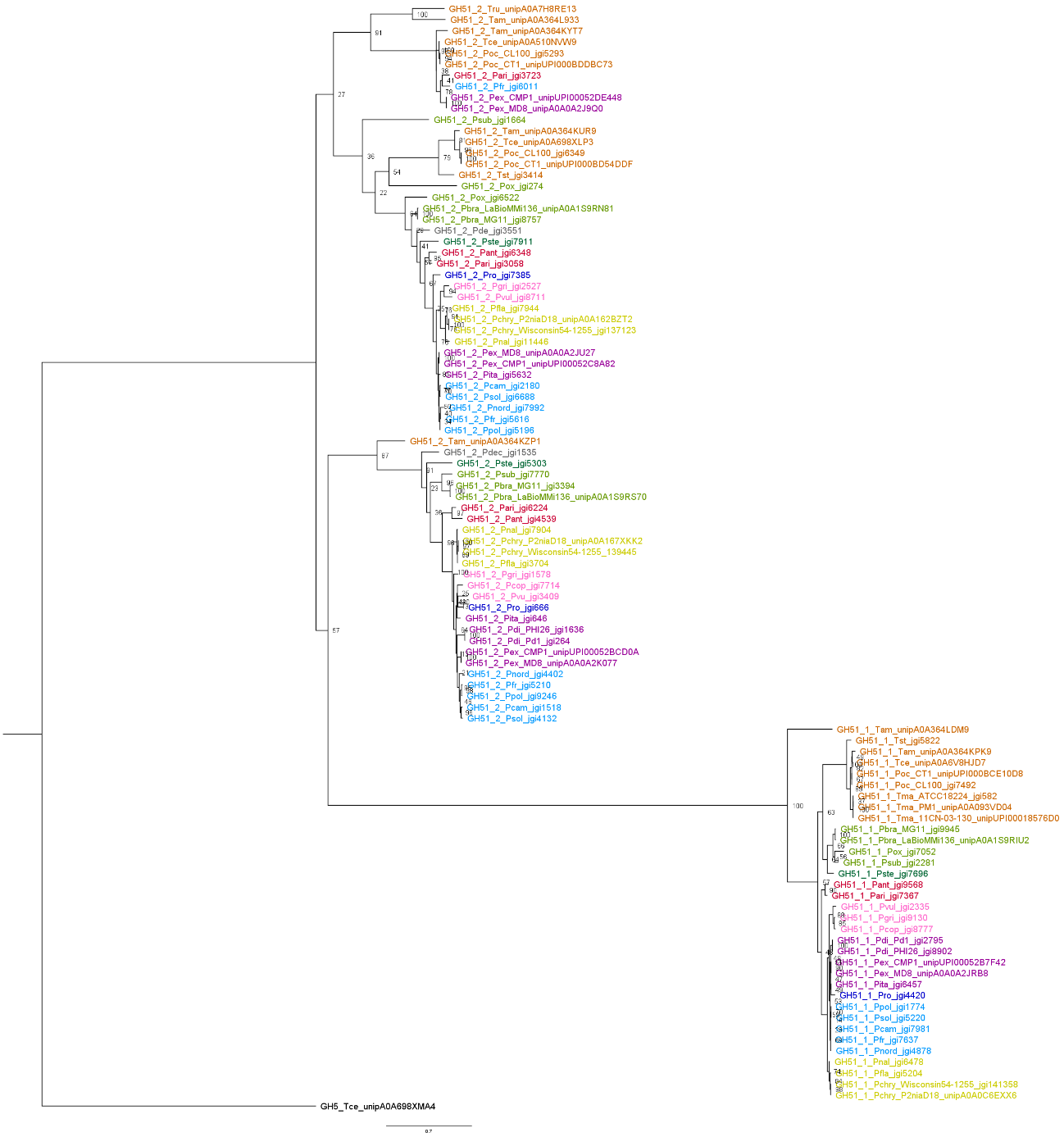


**Figure S2.** Phylogenetic tree of GH51 arabinofuranosidases from *Penicillium* and *Talaromyces*. The root sequence is a GH5 endoglucanase from *Talaromyces celluloyticus* Y-94.


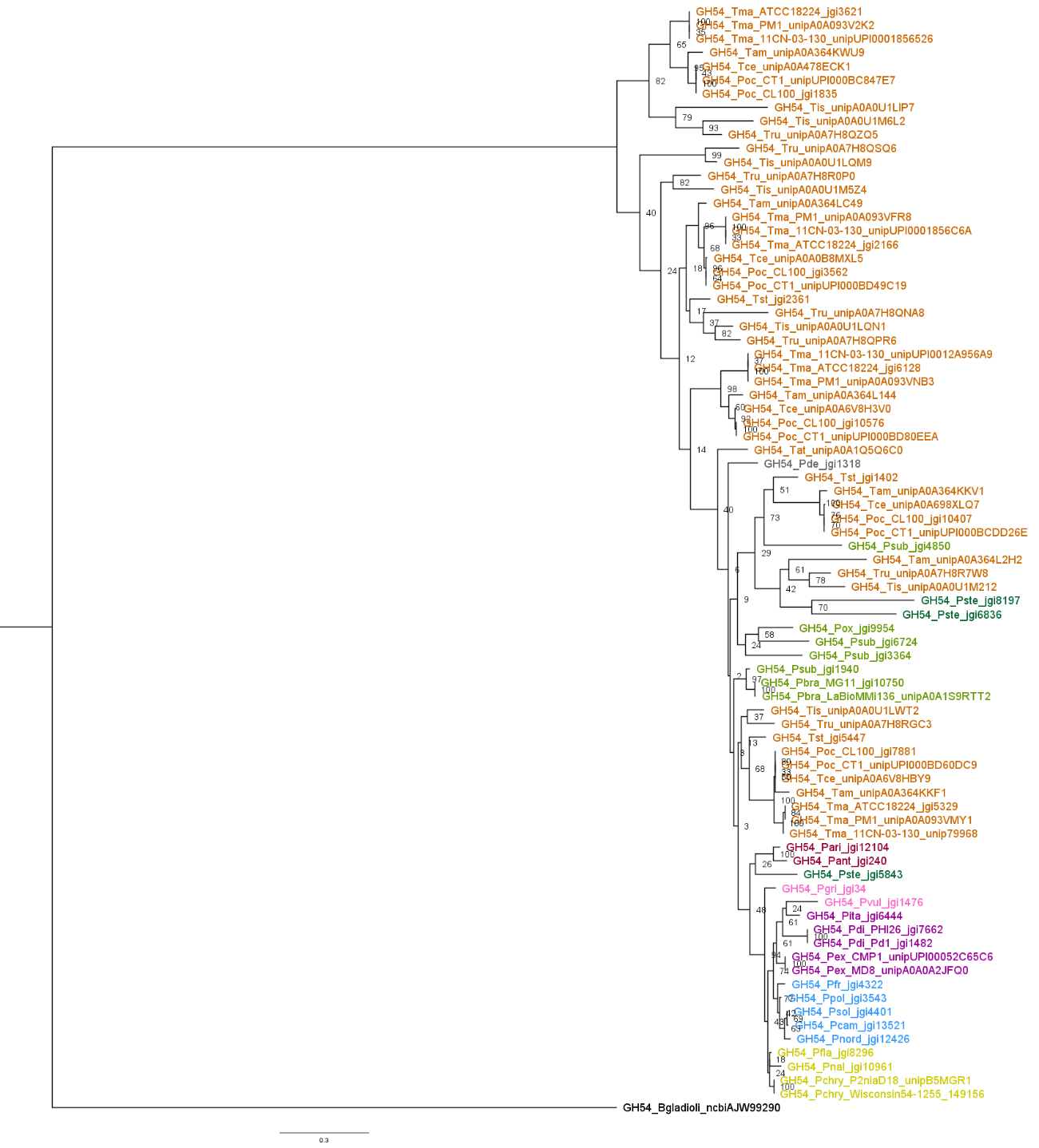


**Figure S3.** Phylogenetic tree of GH54 arabinofuranosidases from *Penicillium* and *Talaromyces.* The root sequence is a GH54 arabinofuranosidase from the bacterium *Burkholderia gladioli*.


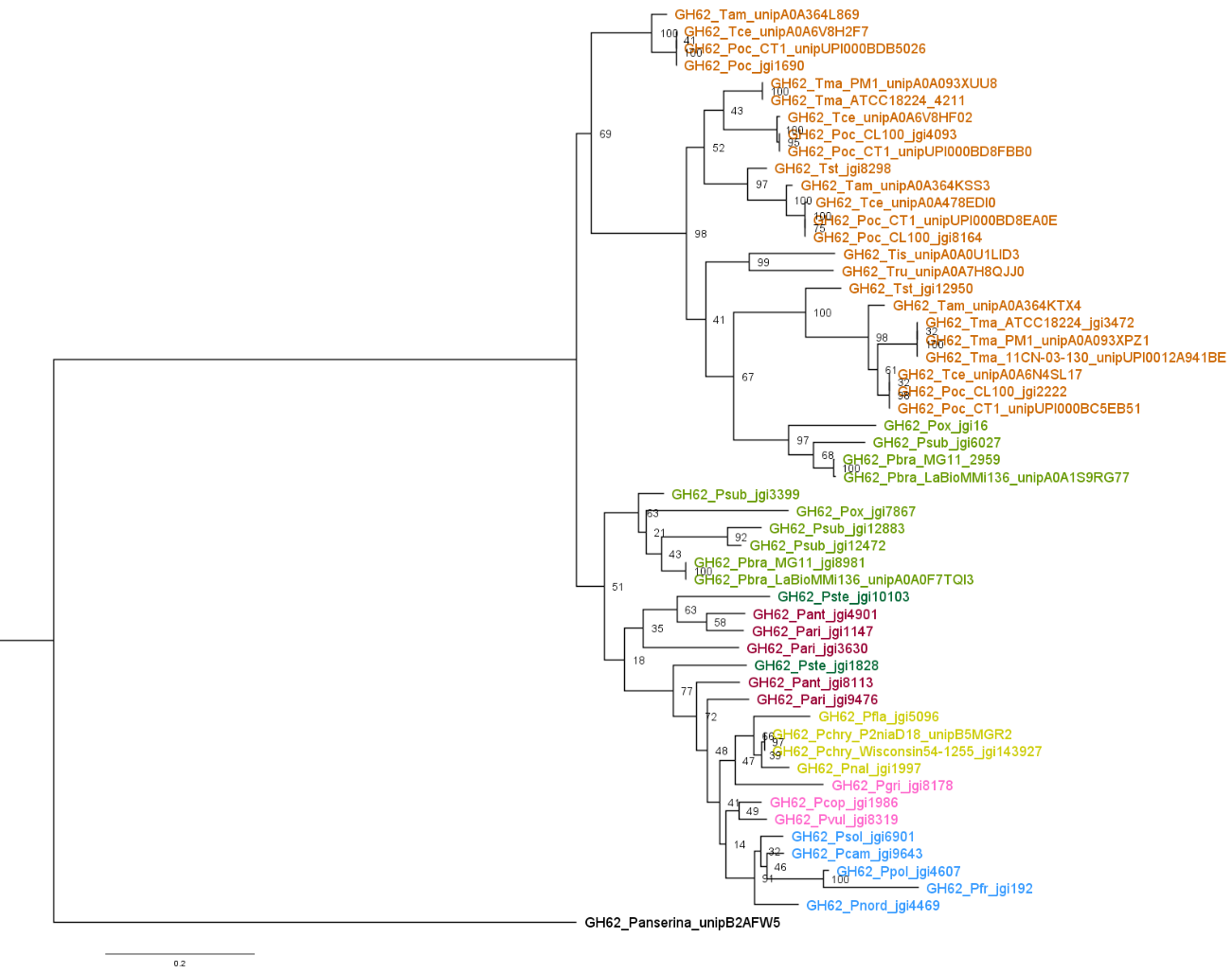


**Figure S4.** Phylogenetic tree of GH62 arabinofuranosidases from *Penicillium* and *Talaromyces*. The root sequence is a GH62 arabinofuranosidase from the fungus *Podospora anserine*.


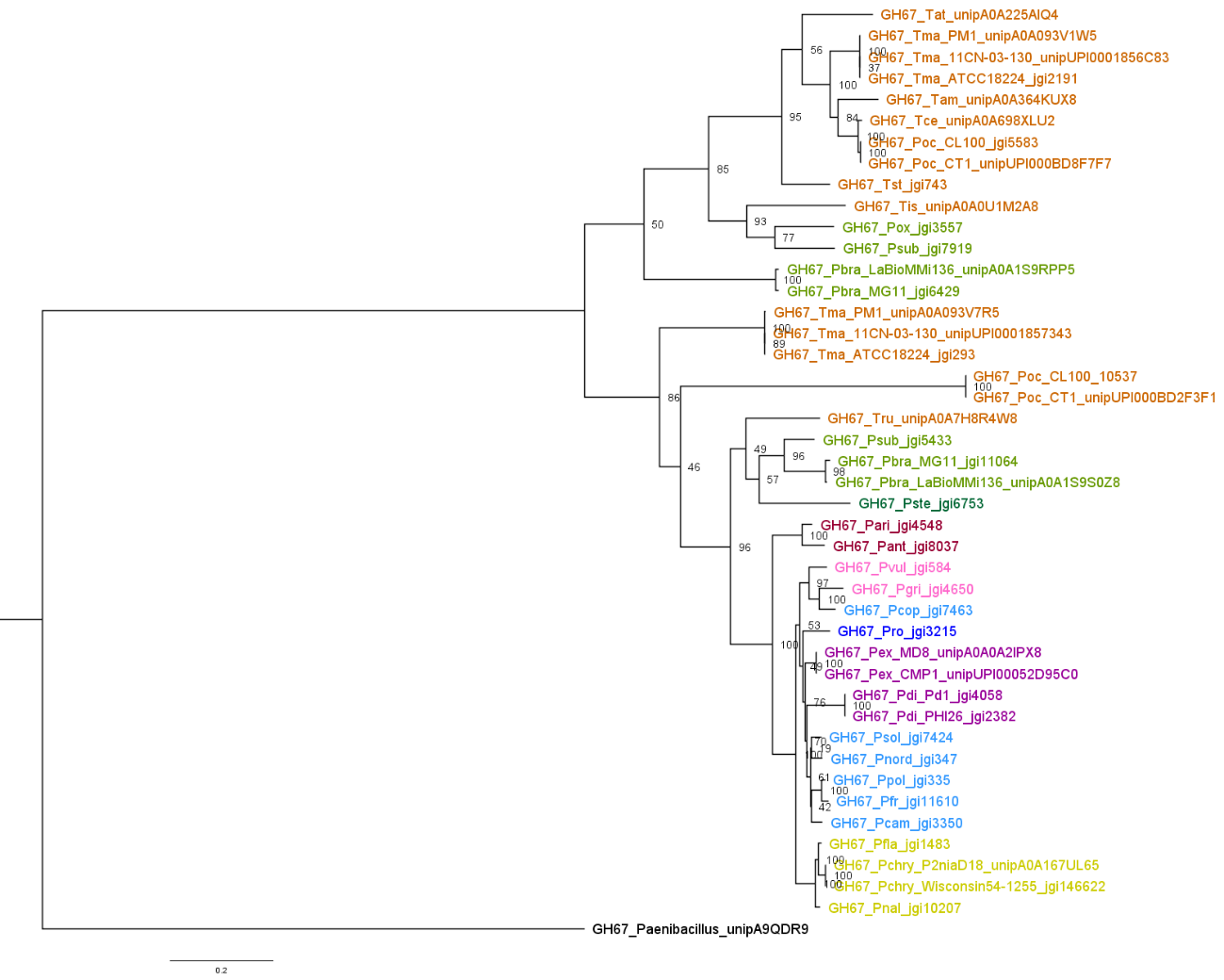


**Figure S5.** Phylogenetic tree of GH67 glucuronidases from *Penicillium* and *Talaromyces*. The root sequence is a GH67 glucuronidase from a *Paenibacillus* sp.


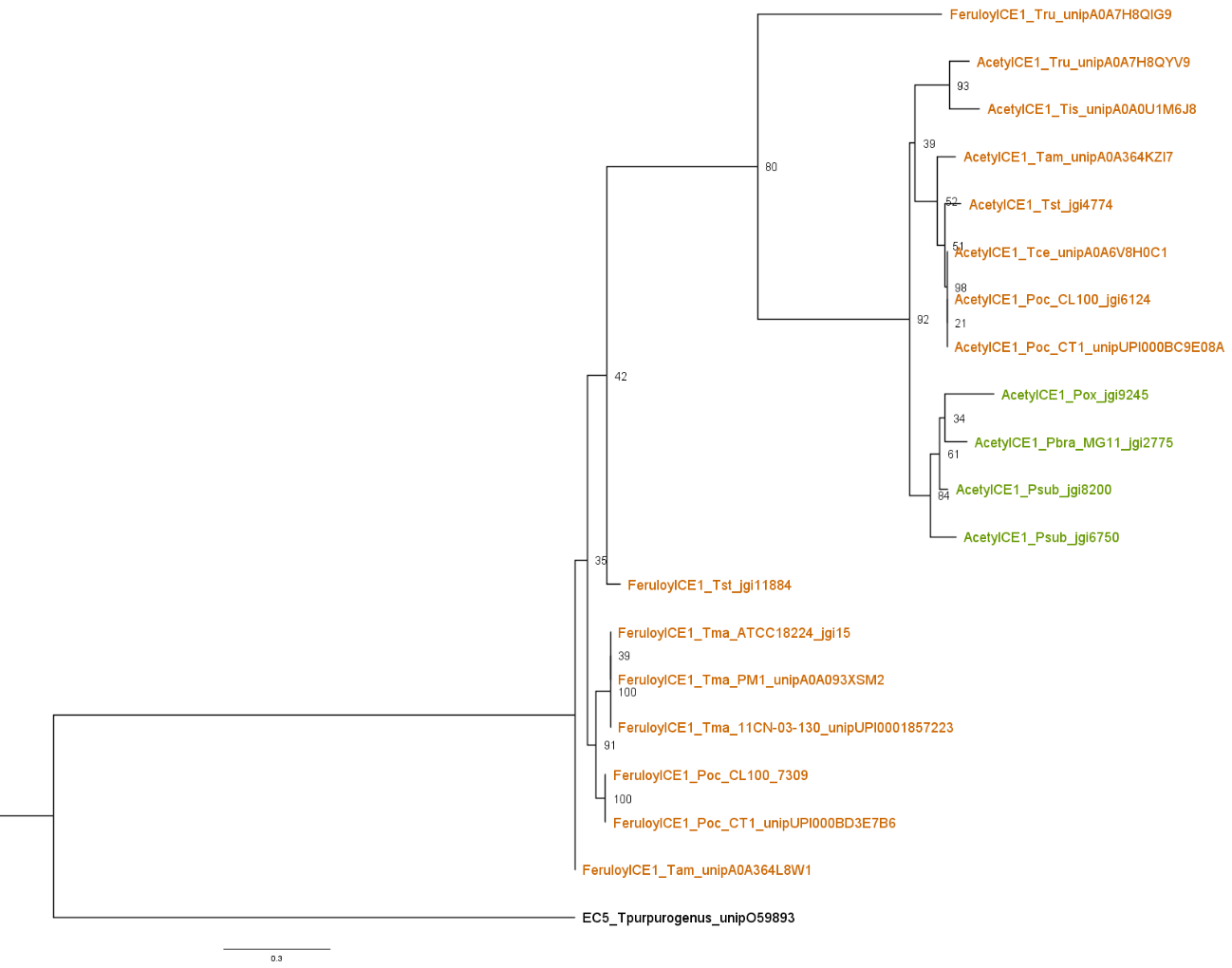


**Figure S6.** Phylogenetic tree of CE1 enzymes (acetylxylan esterases and feruloyl esterases) from *Penicillium* and *Talaromyces*. The root sequence is a CE5 acetylxylan esterase from the fungus *Talaromyces purpurogenus*.


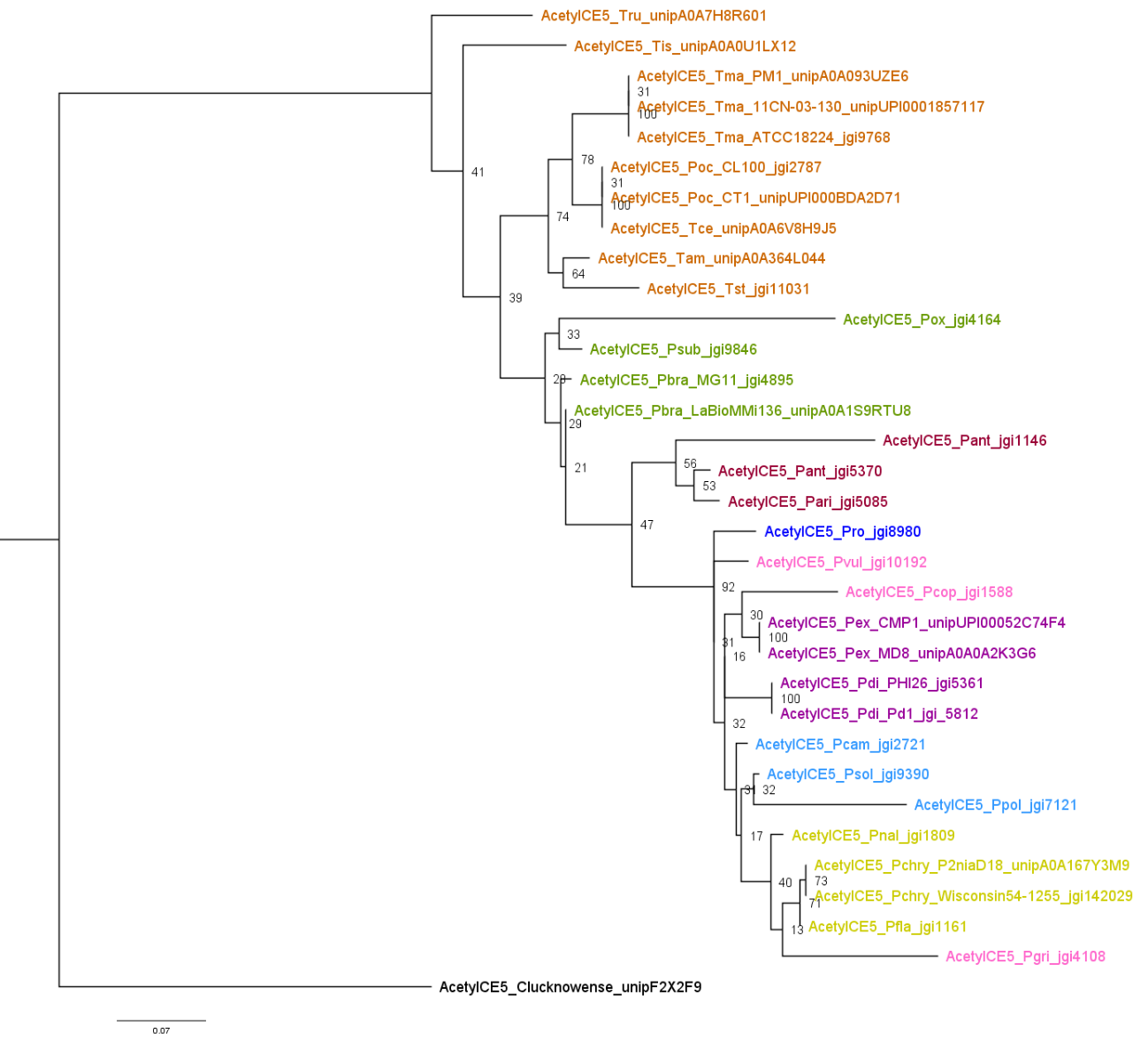


**Figure S7.** Phylogenetic tree of CE5 acetylxylan esterases from *Penicillium* and *Talaromyces*. The root sequence is a CE5 acetylxylan esterase from the fungus *Chrysosporium lucknowense*.
